# Cerebellar ataxia-onset ALS with *SOD1* D91A mutation: a rare phenotype

**DOI:** 10.3389/fneur.2026.1889931

**Published:** 2026-08-04

**Authors:** Denis V. Shevchuk, Evgeny P. Nuzhnyi, Ekaterina Yu. Fedotova, Margarita V. Ershova, Ivan V. Minaev, Georgy A. Anikin, Arina R. Protsenko, Natalia Yu. Abramycheva, Maria N. Zakharova, Sergey N. Illarioshkin

**Affiliations:** Russian Center of Neurology and Neurosciences, Moscow, Russia

**Keywords:** amyotrophic lateral sclerosis, Asp91Ala, cerebellar ataxia, D91A, genotype–phenotype correlation, motor neuron disease, *SOD1*

## Abstract

**Background:**

Amyotrophic lateral sclerosis (ALS) associated with mutations in the superoxide dismutase 1 (*SOD1*) gene is recognized for phenotypic variability, yet cerebellar ataxia as a presenting feature has been reported only in isolated cases.

**Methods:**

We describe four unrelated patients: three men and one woman, aged 35 to 49 years at symptom onset, who carried the *SOD1* D91A (p.Asp91Ala) mutation. Two patients were heterozygous and two homozygous for the D91A variant. Clinical, neuroimaging, electrophysiological and genetic data were reviewed.

**Results:**

All patients presented with progressive gait ataxia as their initial and predominant symptom, with upper and lower motor neuron signs emerging months to years later and ultimately meeting criteria for ALS. Diagnostic latencies from ataxia onset to recognition of ALS ranged from 3 to 9 years. Cerebellar signs included gait and limb ataxia, dysmetria, intention tremor, and oculomotor abnormalities; neuroimaging revealed mild cerebellar atrophy only in one case, and electrophysiological evidence of lower motor neuron involvement was often limited at initial assessment. To our knowledge, this is the largest reported case series of patients homogeneous for a single *SOD1* mutation and a shared cerebellar ataxia-onset ALS phenotype.

**Conclusion:**

The findings expand the clinical spectrum of *SOD1*-associated ALS and underscore the importance of *SOD1* genetic testing in patients with progressive adult-onset ataxia of undetermined origin, particularly given the emerging availability of *SOD1*-targeted therapies.

## 1 Introduction

Amyotrophic lateral sclerosis (ALS) is a progressive neurodegenerative disorder defined by the combined involvement of upper and lower motor neurons, leading to weakness, spasticity, muscle wasting, and respiratory failure. Although this classical definition remains clinically useful, the phenotypic boundaries of ALS have expanded considerably, with frontotemporal dysfunction, autonomic disturbances, sensory abnormalities, and extrapyramidal features now acknowledged in subsets of patients (1, 2).

Pathogenic variants in the superoxide dismutase 1 (*SOD1*) gene were the first identified genetic cause of ALS (3) and remain central to our understanding of the disease. *SOD1* variants account for approximately 12–23% of familial and 1–7% of apparently sporadic ALS cases, with more than 230 individual pathogenic variants cataloged to date (1, 4). Significant genotype–phenotype correlations have emerged across these variants, though intrafamilial variability is also well documented (5). The D91A (p.Asp91Ala; c.272A>C) variant occupies a unique position in this genetic landscape. In Scandinavian populations, D91A behaves predominantly as a recessive allele: homozygous carriers develop a slowly progressive form of ALS, typically with ascending leg-onset weakness, while heterozygous carriers generally remain unaffected (6, 7). This pattern is attributed to a cis-acting protective factor on the recessive haplotype that may modulate disease penetrance (8). Outside Scandinavia, however, the same variant can cause ALS in heterozygous carriers, with considerable phenotypic diversity (4, 5, 9). The D91A variant is the most prevalent and geographically widespread *SOD1* mutation globally, found across ethnic populations from Mongolia through Siberia and throughout Europe (1, 4).

Cerebellar ataxia is not a syndrome traditionally associated with ALS. Although neuropathological studies have documented subclinical cerebellar degeneration in the end-stage of ALS, including spinocerebellar tract involvement and dentate nucleus pathology (10), and transgenic *SOD1* mouse models have demonstrated Purkinje cell calbindin down-regulation and cerebellar architectural defects (11), clinically manifest cerebellar signs during life have been reported only rarely (2, 12). Isolated case reports have described cerebellar ataxia accompanying *SOD1*-associated amyotrophic lateral sclerosis: Yasser et al. (12) in a patient with the E100K mutation; Marsili et al. (13) with a novel R116H variant and a co-existing ceruloplasmin gene variant proposed as a phenotypic modifier; Sequeira et al. (14) in a patient with homozygous D91A who developed motor neuron signs only after 7 years and died 9 years after onset; and our group, who first reported cerebellar ataxia associated with the D91A variant in the heterozygous state (15).

These scattered observations raise a question that isolated reports cannot answer: does the D91A mutation carry a specific predisposition to cerebellar involvement, or are such presentations coincidental? We now present four patients, including the index case previously reported by Nuzhnyi et al. (15), all carrying the *SOD1* D91A mutation, in whom progressive cerebellar ataxia was the first and predominant manifestation of ALS. The cases expand the phenotypic spectrum of *SOD1*-associated ALS, illustrate the diagnostic delay inherent in an atypical presentation, and have implications for the evaluation of progressive adult-onset ataxia, particularly given the availability of *SOD1*-targeted antisense oligonucleotide therapies (1, 16, 17).

## 2 Methods

**Figure 1:**
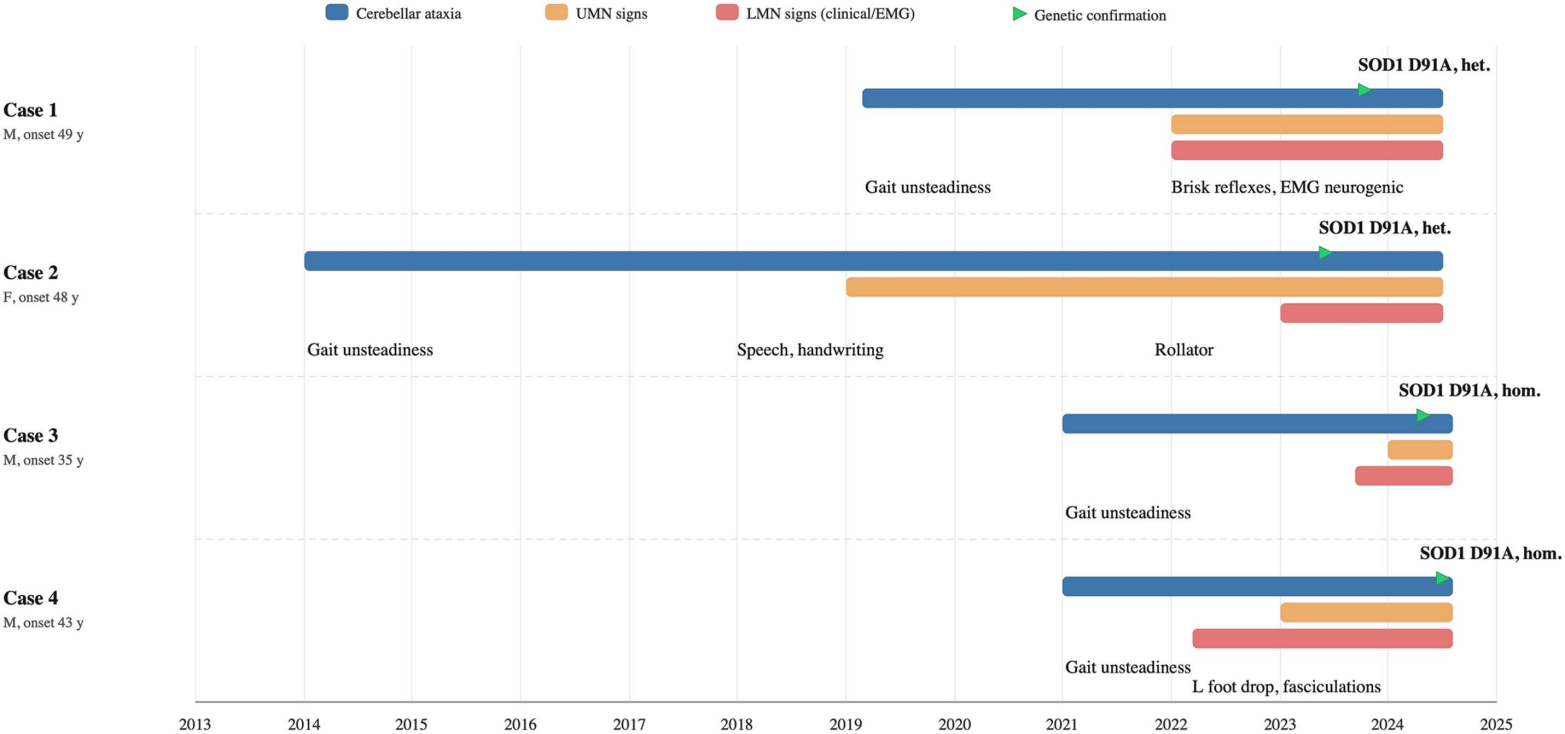
Clinical timeline of four patients with *SOD1* D91A cerebellar ataxia-onset ALS. Temporal relationship between cerebellar ataxia onset, emergence of motor neuron signs, and genetic confirmation. Horizontal bars show duration of each clinical feature from first documentation to last evaluation. Green triangle marks the date of *SOD1* genetic confirmation.

We present a case series of four patients evaluated at the Russian Center of Neurology and Neurosciences, Moscow, Russia. All patients underwent a standardized diagnostic workup for progressive adult-onset ataxia, including comprehensive neurological examination, brain and spinal cord MRI, nerve conduction studies and needle electromyography (EMG), cerebrospinal fluid analysis, screening for acquired causes of ataxia (serum folate, vitamin B12, and vitamin E levels, thyroid function tests, anti-gliadin, anti-GAD, and extended paraneoplastic and autoimmune cerebellar antibody panels covering both classical onconeural antigens and neuronal surface antigens), genetic testing for the most common spinocerebellar ataxias (SCA1, 2, 3, 6, 7, 8, 17, CANVAS and Friedreich ataxia), and whole-exome or panel-based sequencing that included *SOD1*, with identified variants confirmed by Sanger sequencing (Supplementary Figure S1). Additional investigations were performed as clinically indicated and are detailed in the individual case descriptions below. The complete results of the panel used to exclude alternative hereditary and acquired causes of ataxia are summarized in Supplementary Table S1; clinical characteristics are summarized in Table 1; the temporal relationship between ataxia onset and motor neuron signs is depicted in Figure 1.

**Table 1:** Comparative clinical characteristics of four patients with *SOD1* D91A cerebellar ataxia-onset ALS.

Characteristic	Case 1	Case 2	Case 3	Case 4
Sex	Male	Female	Male	Male
Age at last evaluation, years	54	58	38	46
Age at symptom onset, years	49	48	35	43
*SOD1* D91A zygosity	Heterozygous	Heterozygous	Homozygous	Homozygous
*ATXN2 CAG repeats*	22/22	22/22	22/22	22/27 (CAA-interrupted)
*C9orf72 G4C2 expansion*	Negative	Negative	Negative	Negative
First symptom	Gait unsteadiness, leg fatigue	Gait unsteadiness	Mild gait unsteadiness	Mild gait unsteadiness
Disease duration at last evaluation, years	~5	~10	~3.5	~3
Time to ALS diagnosis, years	~5	~9	~3	~3
Initial diagnostic hypotheses	Stiff-person syndrome	Spinocerebellar ataxia	Spinocerebellar ataxia	Neurodegenerative disease
Cerebellar signs	Gait and truncal ataxia, dysmetria (FN, HK)	Severe gait ataxia, severe dysmetria, intention tremor, scanning speech, hypermetric saccades	Gait ataxia, dysmetria, intention tremor, titubation, impaired smooth pursuit, square-wave jerks	Gait ataxia, dysmetria, saccadic pursuit, horizontal nystagmus, dysdiadochokinesis
UMN signs	Brisk arm reflexes, elevated jaw reflex, bilateral PMR reflex	Spastic arms, brisk arm reflexes, bilateral Babinski	Bilateral Babinski	Brisk arm reflexes, bilateral Babinski, snout reflex, bilateral PMR reflex
LMN signs (clinical)	R leg weakness (proximal 3-4/5), hypotonia R leg	Mild proximal R arm and distal L hand weakness (4.5/5)	Distal hand weakness, proximal leg weakness, muscle atrophy, fasciculations	Proximal leg weakness (4/5), absent ankle reflexes, steppage L
EMG findings (latest)	Denervation lumbosacral level (S1 myotome)	Mild reinnervation, no spontaneous activity	No convincing peripheral MN involvement	Active denervation L2-L4, S1-S2; multiple levels fasciculations
Brain MRI	WM hyperintensities (Fazekas 1), Rathke cleft cyst; no cerebellar atrophy	WM hyperintensities (Fazekas 1), no cerebellar atrophy	Rostral vermis atrophy	WM hyperintensities (Fazekas 1)
FVC, % predicted	92%	62.4%	93%	97.7%
Bulbar involvement	Mild dysphonia	Severe dysarthria, moderate dysphagia	None	None
Sensory findings	Normal	Normal	Painful hyperesthesia (feet)	Stocking hypoesthesia, hyperalgesia (feet)
Family history (neurodegenerative)	Negative	Negative	Paternal side unknown	Sister: ALS, homozygous *SOD1*-D91A
Mobility at last evaluation	Walks with bilateral support	Rollator-dependent, cannot stand unsupported	Walks independently, ataxic gait with foot slapping	Walks independently, stamping gait on wide base

ALS, amyotrophic lateral sclerosis; CNS, central nervous system; CSF, cerebrospinal fluid; EMG, electromyography; FN, finger-to-nose; FVC, forced vital capacity; HK, heel-to-knee; L, left; MN, motor neuron; MND, motor neuron disease; PMR, palmomental reflex; MRI, magnetic resonance imaging; R, right; UMN, upper motor neuron; LMN, lower motor neuron, WM, white matter.

Cases 1, 2 and 3 were unrelated individuals of Slavic (Russian) ancestry originating from different geographic regions of the Russian Federation, with no known consanguinity or shared pedigree; the only related individuals in this report are the patient in Case 4 and his affected sister. In addition to the spinocerebellar ataxia panel, all five individuals were screened for the *C9orf72* G4C2 hexanucleotide repeat expansion using fluorescent amplicon-length analysis combined with repeat-primed PCR. *ATXN2* CAG repeat length was determined by PCR amplification of exon 1 with fluorescently labeled primers followed by capillary fragment-length analysis; alleles in the intermediate range were further characterized by Sanger sequencing to resolve the number and position of CAA interruptions within the CAG tract.

## 3 Results

### 3.1 Case 1

A man aged 49 years first noticed intermittent fatigue in both legs. Approximately 1.5 years later, he developed gait unsteadiness and slight right-leg limp, and numbness involving the second and third toes of the right foot. During evaluation at our center approximately 2 years after symptom onset, the differential diagnosis included stiff-person syndrome. The standard ataxia workup was unremarkable. EMG findings were initially limited, showing only isolated fasciculation potentials. Brain MRI (3 years after onset) showed white matter hyperintensities (Fazekas 1), and a Rathke cleft cyst without posterior fossa abnormalities. Cerebrospinal fluid analysis was unremarkable. A subsequent admission raised the possibility of motor neuron disease, but follow-up EMG showed no spread of denervation above the lumbosacral level; spinal MRI excluded myelopathy.

Four years after onset, targeted sequencing revealed a heterozygous pathogenic variant c.272A>C (p.Asp91Ala) in exon 4 of the *SOD1* gene, confirming the diagnosis of *SOD1*-associated amyotrophic lateral sclerosis. Treatment with riluzole in standard dose was initiated.

At the latest evaluation, approximately 5 years after symptom onset (age 54), the neurological examination demonstrated a paretic-ataxic gait on a wide base requiring bilateral support, dysmetria on finger-to-nose and heel-to-knee testing, falling in the Romberg position, mild dysphonia, an elevated jaw reflex, bilateral palmomental reflex, and brisk upper limb reflexes. Proximal right leg weakness was present (hip flexors 4/5, extensors 3/5), with hypotonia in the right leg. Sensory examination was normal. FVC was 92% predicted. Repeat EMG at the latest evaluation demonstrated active denervation in the S1 myotome (right more than left), though data remained insufficient to confirm generalized disease spread. There was no family history of neurodegenerative disease.

### 3.2 Case 2

A woman aged 48 years developed slowly progressive leg weakness and gait unsteadiness. Four years later, handwriting deteriorated, and her speech became less distinct. Inpatient evaluation approximately 5 years after onset included the standard ataxia workup (see above), which was unremarkable; brain MRI showed small-vessel white matter changes but no cerebellar atrophy.

Six years after onset, the patient required unilateral gait support; by 8 years, she used a rollator, with worsening dysarthria and occasional choking. EMG (9 years after onset) revealed reinnervation changes at multiple segmental levels without spontaneous activity. Gene panel sequencing identified a heterozygous pathogenic *SOD1* variant c.272A>C (p.Asp91Ala). This finding established the molecular diagnosis 9 years after symptom onset. The initial characterization of this patient was previously published (15).

At the latest evaluation, 10 years after symptom onset (age 58), the patient exhibited severe dysarthria with scanning speech, moderate dysphagia, a weakened cough reflex, hypermetric vertical and horizontal saccades, spasticity and paratonia more prominent in the lower limbs, markedly hyperactive arm reflexes with expanded reflexogenic zones, reduced leg reflexes, bilateral Babinski signs. Finger-to-nose and heel-to-knee testing demonstrated severe dysmetria with intention tremor bilaterally. She could not stand unsupported and used a rollator for all ambulation. Mild proximal weakness was present in the right arm and distal weakness in the left hand (both 4.5/5). Repeat EMG at the latest evaluation showed stable mild reinnervation changes without spontaneous activity—unchanged from the previous year. FVC was 62.4% predicted; nocturnal pulse oximetry showed a mean SpO2 of 96.7%. Family history was unremarkable for neurological disease.

### 3.3 Case 3

**Figure 2:**
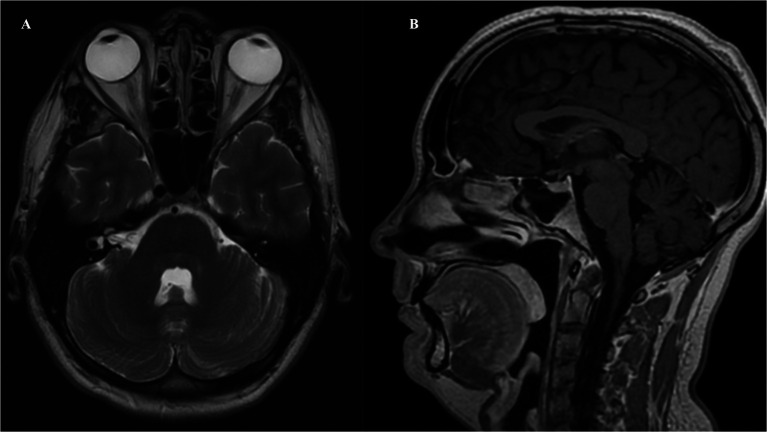
Brain MRI of patient 3. **(A)** Axial T2-weighted image showing mild enlargement of the fourth ventricle. **(B)** Midline sagittal T1-weighted image demonstrating mild atrophy of the rostral cerebellar vermis.

A man aged 35 years, the youngest in the series, noticed intermittent lateral swerving during head turns. One year later, his gait was visibly abnormal. A neurologist diagnosed probable spinocerebellar ataxia. Brain MRI was initially normal but approximately 3 years after onset revealed mild cerebellar vermis atrophy (Figure 2). The standard ataxia workup was unremarkable. Whole-exome sequencing (3 years after onset) identified a homozygous *SOD1* variant, confirmed by Sanger sequencing as c.272A>C (p.Asp91Ala).

At the latest evaluation, approximately 3.5 years after symptom onset (age 38), examination showed hypermetric saccades, mild distal weakness in both hands (4/5, worse on the left), proximal leg weakness (left 4/5, right 4.5/5) with prominent paresis of knee flexors on the left (3/5), reduced muscle tone, muscle atrophy in the thighs and calves, evoked fasciculations in the thigh muscles, absent knee reflexes with reduced ankle reflexes, bilateral Babinski signs. Coordination testing showed mild dysmetria with intention tremor, worse on the right. Truncal titubation was present. Gait was ataxic with foot slapping; tandem walking was impossible and the Romberg test was positive. Video-oculography confirmed a latent leftward nystagmus, impaired smooth pursuit, and an increased frequency of square-wave jerks consistent with cerebellar dysfunction. Needle EMG showed neurogenic motor unit changes in the lower extremities without spontaneous denervation activity and was considered inconclusive for generalized lower motor neuron involvement. FVC was 93% predicted. Painful hyperesthesia was present in the feet. A specialist consultation confirmed a diagnosis of ALS based on Gold Coast criteria (18), with clinical evidence of upper and lower motor neuron involvement at two levels of the cerebrospinal axis. Family history on the paternal side was unknown, and no neurological disease was reported on the maternal side.

### 3.4 Case 4

A man aged 43 years developed mild gait unsteadiness. Six months later, left foot drop appeared with foot slap and inversion. By 2 years after onset, running was impossible, with frequent cramps and fasciculations in the legs. He was evaluated locally and diagnosed with a neurodegenerative disease. The standard ataxia workup was unremarkable except for CSF analysis (3 years after onset), which showed slight protein elevation (0.74 g/L). Brain MRI showed isolated white matter foci without corticospinal tract abnormalities; spinal MRI showed degenerative vertebral changes without myelopathy.

At our center (3 years after onset), EMG revealed active denervation (fibrillation potentials, positive sharp waves) in L2-L4 and S1-S2 myotomes, fasciculation potentials in the gastrocnemius, tibialis anterior, and deltoid muscles, neurogenic motor unit remodeling in all sampled muscles, and axonal sensory neuropathy in the lower extremities. Targeted sequencing confirmed a homozygous *SOD1* variant c.272A>C (p.Asp91Ala)—the same genotype previously identified in his sister, who had been diagnosed with ALS at our center.

At the latest evaluation (age 46), examination showed saccadic smooth pursuit and horizontal gaze-evoked nystagmus in extreme lateral positions. There was no dysarthria, dysphagia, or dysphonia. Proximal leg weakness (4/5, worse on the left) was accompanied by mild spasticity in the lower limbs. Arm reflexes were brisk with expanded reflexogenic zones; knee reflexes were present with thigh adduction, while ankle reflexes were absent bilaterally. The Babinski sign was present bilaterally, together with a positive snout reflex and bilateral palmomental reflex. Coordination testing showed moderate dysmetria with a mild intention component and mild dysdiadochokinesis. The gait was stamping, on a widened base, with mild left-sided steppage. FVC was 97.7% predicted. Sensory examination revealed stocking-distribution hypoesthesia with hyperalgesia in the feet. Family history was notable for a sister with confirmed ALS carrying the homozygous *SOD1* D91A variant. Her disease began with progressive leg weakness and gait unsteadiness at age 42, followed by upper limb weakness 2 years later and bulbar involvement (dysarthria, dysphagia) after 3 years. Brain MRI demonstrated cortical and vermis cerebellar atrophy as well as corticospinal tract signal hyperintensity. At age 46, FVC was 42.5–56.6% predicted.

### 3.5 Synthesis

All four patients followed a remarkably similar trajectory: slowly progressive gait ataxia in adulthood, followed, after a latency of one to several years, by the gradual emergence of upper and lower motor neuron signs (Table 1, Figure 1). In each case, the ataxia dominated the early clinical presentation and directed the diagnostic workup toward spinocerebellar degenerations or acquired cerebellar disorders. There was no anamnestic or clinical evidence of alcohol abuse as a cause of progressive cerebellar ataxia. The interval from ataxia onset to molecular or clinical diagnosis of amyotrophic lateral sclerosis was 3 years in Cases 3 and 4, 5 years in Case 1, and 9 years in Case 2.

The cerebellar features: wide-based ataxic gait, limb dysmetria, intention tremor, oculomotor abnormalities—were strikingly consistent across cases, though they varied in severity. Case 2, with the longest disease duration at the time of evaluation, displayed the most pronounced ataxia and the most significant bulbar involvement (dysarthria, dysphagia) and respiratory compromise (FVC 62%). Case 3 was the only patient with documented truncal titubation and with neuroimaging evidence of mild cerebellar vermis atrophy. Upper motor neuron signs were present in all four patients at latest evaluation—hyperreflexia with expanded reflexogenic zones in the arms, Babinski signs, and spasticity, while lower motor neuron features (weakness, atrophy, fasciculations, and denervation on EMG) were variably distributed and often relatively modest in proportion to the clinical disability. Two patients (Cases 1 and 2) were heterozygous carriers of the D91A variant, while two (Cases 3 and 4) were homozygous.

Genetic findings: no pathogenic *C9orf72* hexanucleotide repeat expansion was detected in any of the five individuals tested (Table 1; Supplementary Table S1). *ATXN2* CAG repeat sizing revealed two normal-length alleles (22/22) in Cases 1, 2 and 3. By contrast, the two homozygous D91A siblings: Case 4 and his affected sister—each carried one normal (22-repeat) and one intermediate-length (27-repeat) allele (genotype 22/27). Sanger sequencing of the expanded allele confirmed 27 CAG repeats in both siblings and demonstrated an identical interrupted configuration, with CAA interruptions at codons 9, 14 and 19 of the repeat tract (Supplementary Figure S2A,B). The intermediate *ATXN2* allele thus co-segregated with the homozygous D91A genotype within this family, whereas the three unrelated D91A patients carried only normal-length *ATXN2* alleles.

## 4 Discussion

### 4.1 Cerebellar involvement in *SOD1*-ALS

The four patients described here share a clinical pattern that is internally consistent and unusual in the published ALS literature: all presented with progressive cerebellar ataxia as the first and predominant manifestation of *SOD1*-associated ALS. The uniform presence of the D91A variant, combined with the shared phenotypic signature, suggests a mutation-specific predisposition to cerebellar involvement.

Cerebellar pathology is not uncommon in ALS. Neuropathological studies have demonstrated Purkinje cell loss, dentate nucleus degeneration, and cerebellar white matter gliosis in subsets of patients (10, 12), and the SOD1 protein is ubiquitously expressed, including in cerebellar neurons. Misfolded SOD1 aggregates have been identified in cerebellar tissue in both human autopsy material and transgenic mouse models (2, 11, 19). Martinelli et al. (2) systematically cataloged extramotor manifestations in *SOD1*-ALS, including cerebellar, extrapyramidal, sensory, and cognitive features. Yet clinically overt ataxia at presentation remains sufficiently rare to steer the diagnostic process away from amyotrophic lateral sclerosis.

Only a handful of cases of *SOD1*-ALS with predominant cerebellar ataxia have been reported. Yasser et al. (12) described a patient with *SOD1* E100K variant who exhibited prominent cerebellar features alongside amyotrophy, though motor neuron signs were present early in the course. Marsili et al. (13) reported a patient with *SOD1* R116H and a concomitant ceruloplasmin gene variant, in whom a 19-year progressive decline combined cerebellar ataxia with late-emerging motor neuron disease and MRI cerebellar atrophy. Sequeira et al. (14) described a patient homozygous for *SOD1* D91A who developed motor neuron signs only 7 years after the initial presentation of cerebellar ataxia and died 9 years after disease onset, with diffuse cortical and cerebellar atrophy on the final brain MRI. The present series, which includes the index case (15) alongside three additional patients, provides the first opportunity to compare multiple patients with the same *SOD1* variant and a shared cerebellar phenotype.

A common thread across these reports and our series is the prolonged diagnostic delay. In our patients, the average interval from ataxia onset to recognition of ALS exceeded 4 years, reaching 9 years in Case 2. This far exceeds the typical 10-to-16-month diagnostic latency reported in population-based ALS studies (20, 21) and reflects the misdirection created by an ataxia-dominant presentation, which consistently directed the workup toward inherited spinocerebellar degenerations and acquired cerebellar disorders.

### 4.2 D91A phenotypic heterogeneity and genetic modifiers

The present series adds a new dimension to D91A variability. Two heterozygous carriers (Cases 1 and 2) demonstrate that cerebellar ataxia-onset disease is possible in the heterozygous state in a non-Scandinavian population, where the protective cis-acting factor associated with the recessive haplotype may be absent (8, 9). Two homozygous patients (Cases 3 and 4) presented with ataxia rather than the ascending leg weakness typically described for this genotype (6, 7). This divergence may reflect additional genetic and epigenetic modifiers, environmental factors, or stochastic variability in *SOD1*-mediated neurodegeneration. In silico modeling studies have shown that the structural impact of individual *SOD1* mutations does not fully predict clinical severity or phenotypic features, supporting the role of modifying factors (5).

Other modifiers remain plausible: Marsili et al. (13) proposed a ceruloplasmin variant as a modifier in their *SOD1*-ataxia patient, and Sequeira et al. (14) highlighted intrafamilial variability in a family with *SOD1* G13R variant, where a proband presented with classical ALS while his father developed cerebellar ataxia with the same mutation. The familial aggregation in our series is particularly informative: Case 4 and his sister both carry homozygous D91A variant, and both exhibit cerebellar involvement. The sister, evaluated at our institution, presented with ascending leg-onset weakness followed by upper limb and bulbar involvement, but brain MRI also demonstrated cortical and vermis cerebellar atrophy, and clinical examination revealed dysmetria and nystagmus. Although her cerebellar findings were initially attributed to prior chemotherapy and radiation therapy for rectal cancer (2015), the concordance with her brother’s cerebellar ataxia-onset phenotype in the context of an identical *SOD1* genotype makes a *SOD1*-mediated mechanism more plausible. This intrafamilial concordance for cerebellar involvement strengthens the hypothesis that the D91A variant carries a specific predisposition to cerebellar pathology. Taken together, these observations point to a cerebellar phenotype shaped by the primary *SOD1* mutation and additional genetic or epigenetic factors.

A further potential modifier emerged from analysis of the *ATXN2* locus. Intermediate-length *ATXN2* polyglutamine expansions, commonly defined as 27 to 33 repeats, are the strongest established polygenic risk factor for amyotrophic lateral sclerosis and act, at least in part, by enhancing TDP-43 toxicity and pathology (22–24). In our series, the three unrelated D91A patients carried normal-length *ATXN2* alleles (22/22), whereas Case 4 and his affected sister, both homozygous for D91A and both showing cerebellar involvement, additionally carried an intermediate-length 27-repeat allele with an identical CAA-interrupted configuration (interruptions at codons 9, 14 and 19). Notably, ALS-associated intermediate *ATXN2* expansions are characteristically CAA-interrupted, in contrast to the pure CAG expansions that cause spinocerebellar ataxia type 2, and the number and position of these interruptions are thought to modulate repeat stability and RNA-mediated toxicity (25–27). Although a 27-repeat allele lies at the lower boundary of the intermediate range and its individual pathogenic weight is uncertain, its co-segregation with the homozygous D91A genotype in the two siblings with the most clearly familial cerebellar phenotype raises the possibility that *ATXN2* acts as an additional genetic modifier in this branch of the family. Because the unrelated patients with normal *ATXN2* alleles displayed the same cerebellar ataxia-onset phenotype, *ATXN2* cannot account for the cerebellar presentation across the series. Rather, the *SOD1* D91A variant remains the primary determinant, potentially compounded by an *ATXN2*-related modifier effect in the sibling pair. Co-occurrence of intermediate *ATXN2* expansions with primary ALS-associated genotypes has been reported previously (28, 29). More broadly, *ATXN2* is increasingly recognized as a powerful modulator of neurological and neurodegenerative disorders, including a role for *de novo* and intermediate-length variants in modifying ALS risk (30, 31).

### 4.3 Electrophysiological and neuroimaging features

A recurring finding is the discrepancy between prominent cerebellar and upper motor neuron signs and relatively limited electrophysiological evidence of lower motor neuron involvement, particularly in the early and middle stages of disease. In Case 2, despite 9 years of progressive neurological deterioration with severe dysarthria, dysphagia, and an FVC of 62%, EMG showed only mild reinnervation without spontaneous activity across two examinations a year apart. Case 3 met Gold Coast criteria clinically, yet EMG at our center was inconclusive for peripheral motor neuron involvement. Only Case 4, at a relatively early clinical stage, showed florid denervation. This pattern raises the question of whether D91A-associated cerebellar ataxia-onset disease preferentially affects the corticospinal and cerebellar systems, including the spinocerebellar tracts, with relative sparing of lower motor neurons, or whether the lower motor neuron component develops more slowly and is detected only with prolonged follow-up. The former possibility would align with neuropathological observations that some *SOD1* mutations produce a distribution of aggregate deposition not restricted to anterior horn cells (2, 10). Studies using patient-derived induced pluripotent stem cells, including work from our institution on *SOD1*-ALS patients, have demonstrated mutation-specific patterns of motor neuron vulnerability *in vitro* (19), but whether cerebellar neurons show differential susceptibility to D91A-mediated toxicity remains to be investigated.

Brain MRI was unremarkable in three of the four patients, showing at most nonspecific vascular white matter changes. The exception was Case 3, in whom MRI demonstrated only mild cerebellar vermal atrophy. The absence of prominent cerebellar atrophy despite the obvious signs of ataxia is consistent with observation of Sequeira et al. (14) where the patient developed diffuse cerebellar atrophy only in later stages of the disease. Clinical cerebellar dysfunction in neurodegenerative disease can precede detectable volume loss by years, and conventional MRI sequences may lack sensitivity for early changes (32). In addition, the absence of cerebellar atrophy on MRI may in part reflect selective involvement of the spinocerebellar tracts and dentate nucleus rather than diffuse cerebellar degeneration, as documented in neuropathological and neuroimaging studies of *SOD1*-ALS (7, 33, 34). Spinal cord MRI in our patients did not show overt cord atrophy, but conventional imaging cannot reliably detect tract-specific changes, and this remains a limitation of our study.

### 4.4 Diagnostic and therapeutic implications

In patients with progressive adult-onset cerebellar ataxia and a negative standard workup for spinocerebellar ataxias, acquired cerebellar disorders, and structural lesions, the emergence of even subtle upper or lower motor neuron signs should prompt consideration of a cerebellar ataxia-onset variant of *SOD1*-ALS. We propose that *SOD1* genetic testing should be incorporated into the diagnostic algorithm for unexplained progressive cerebellar ataxias even with mild upper and/or lower motor neuron signs especially in the absence or minor cerebellar atrophy (15).

This recommendation carries particular weight in the current therapeutic landscape. Tofersen, an intrathecally administered antisense oligonucleotide designed to reduce SOD1 protein expression, has shown meaningful biomarker responses, including reduction of CSF SOD1 and plasma neurofilament light chain concentrations, and clinical benefit in *SOD1*-ALS patients in the Phase 3 VALOR trial and its open-label extension (16, 17), and received accelerated approval by the US FDA in April 2023. Identifying the correct molecular diagnosis in patients with atypical presentations is therefore no longer merely of theoretical interest—it has direct therapeutic consequences. The availability of patient-derived cellular models, such as the iPSC lines generated from Russian *SOD1*-ALS patients by Chestkov et al. (19), further underscores the translational value of accurate genotyping in this population.

### 4.5 Strengths and limitations

The main strength of this series is its homogeneity: four patients with the same *SOD1* mutation and a shared cerebellar ataxia-onset phenotype, allowing direct comparison of clinical trajectories across both zygosity states. Furthermore, the intra-familial concordance for cerebellar involvement in Case 4 and his affected sister, both homozygous D91A carriers, provides within-family evidence that the cerebellar phenotype is linked to the *SOD1* genotype rather than to coincidental environmental or stochastic factors.

Limitations of our study include the absence of neuropathological confirmation of cerebellar involvement in these living patients, the partly retrospective design, which limits the granularity of temporal details and precluded the use of standardized clinical scales (ALSFRS-R, SARA), and the small sample size, which limits generalizability. Prospective studies incorporating advanced neuroimaging (voxel-based morphometry, diffusion tensor imaging of cerebellar pathways) and longitudinal neurophysiology will be needed to characterize the natural history of cerebellar ataxia-onset *SOD1*-ALS.

## 5 Conclusion

We describe four patients with the *SOD1* D91A mutation who presented with progressive cerebellar ataxia as the initial manifestation of amyotrophic lateral sclerosis. The series includes both heterozygous and homozygous carriers and encompasses a range of disease severities and durations. These cases expand the phenotypic spectrum of *SOD1*-associated ALS and demonstrate that cerebellar ataxia-onset presentations cause substantial diagnostic delay. Clinicians evaluating patients with progressive adult-onset ataxia of undetermined cause should consider *SOD1*-ALS in the differential diagnosis, particularly when upper or lower motor neuron signs are present. *SOD1* genetic testing should be included in the diagnostic algorithm for such patients, especially now that *SOD1*-targeted gene therapies are becoming available.

## Data Availability

The original contributions presented in the study are included in the article/Supplementary material, further inquiries can be directed to the corresponding author.

## Supplementary material

SUPPLEMENTARY FIGURE S1 Sanger sequencing confirmation of the *SOD1* c.272A>C (p.Asp91Ala) variant. Representative forward-strand electropherograms of SOD1 exon 4 spanning codon 91 (highlighted; bracket below). Top, wild-type reference control showing the unaltered codon GAC (Asp); middle, a heterozygous carrier (Cases 1 and 2), in which the c.272A>C substitution produces a double peak (A/C) at the second codon position [G(A/C)C]; bottom, a homozygous carrier (Cases 3 and 4 and the affected sister), showing complete replacement by GCC (Ala). The orange band and arrowhead mark the variant position (c.272). Nucleotides are coloured by the standard convention (A, green; C, blue; G, black; T, red).

SUPPLEMENTARY FIGURE S2 *ATXN2* intermediate-length allele in the two affected siblings. **(A)** *ATXN2* CAG repeat-length analysis (fragment sizing) in Case 4 and his affected sister: each carries one normal (22-repeat; ≈138 bp) and one intermediate-length (27-repeat; ≈154 bp) allele (genotype 22/27). **(B)** *ATXN2* CAG-tract structure determined by Sanger sequencing (schematic): blue squares, CAG codons; orange squares, CAA interruptions. The intermediate 27-repeat allele shared by both siblings shows an identical interrupted configuration [(CAG)_8_ CAA (CAG)_4_ CAA (CAG)_4_ CAA (CAG)_8_], with interruptions at codons 9, 14 and 19; the normal allele is shown in the common reference configuration. The original electropherograms and chromatograms are available from the corresponding author on request.
