# Supplementary material for: Cerebellar ataxia-onset ALS with SOD1 D91A mutation: a rare phenotype
Source: Front Neurol. 2026 Aug 4;17:1889931. doi: 10.3389/fneur.2026.1889931 (PMC13481206; doi:10.3389/fneur.2026.1889931)
Supplement: Supplementary file 1 [file Data_Sheet_1.pdf]

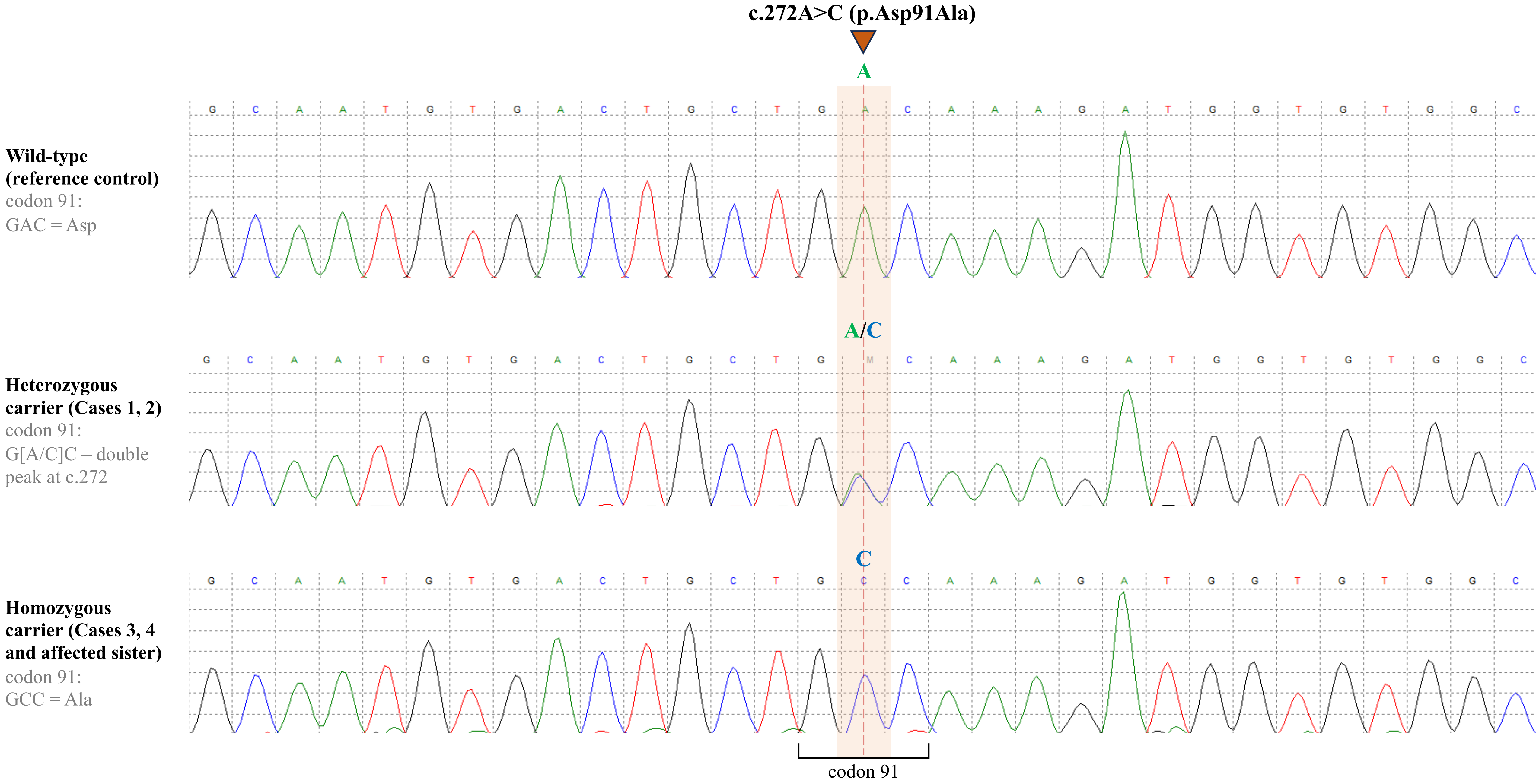

c.272A>C (p.Asp91Ala)
A
G C A A T G T G A C T G C T G A C A A A G A T G G T G T G G C
Wild-type (reference control)
codon 91:
GAC = Asp
A/C
G C A A T G T G A C T G C T G M C A A A G A T G G T G T G G C
Heterozygous carrier (Cases 1, 2)
codon 91:
G[A/C]C – double peak at c.272
C
G C A A T G T G A C T G C T G C C A A A G A T G G T G T G G C
Homozygous carrier (Cases 3, 4 and affected sister)
codon 91:
GCC = Ala
codon 91
