# Supplementary material for: Cerebellar ataxia-onset ALS with SOD1 D91A mutation: a rare phenotype
Source: Front Neurol. 2026 Aug 4;17:1889931. doi: 10.3389/fneur.2026.1889931 (PMC13481206; doi:10.3389/fneur.2026.1889931)
Supplement: Supplementary file 2 [file Data_Sheet_2.pdf]

## A *ATXN2* CAG repeat-length analysis (fragment sizing)

Case 4 and his affected sister: each carries a normal 22-repeat (≈138 bp) and an intermediate 27-repeat (≈154 bp) allele (genotype 22/27).

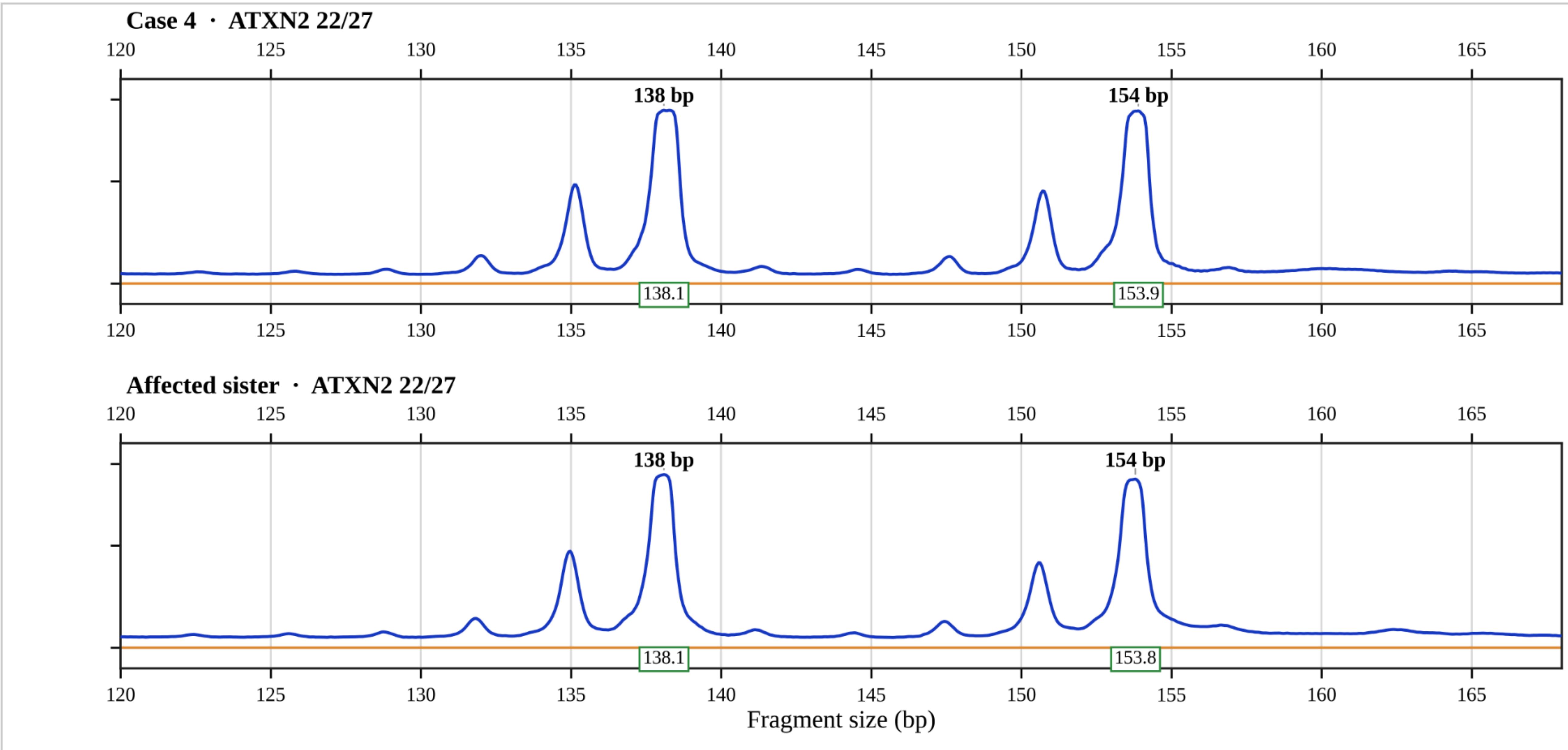


## B *ATXN2* CAG-tract structure (Sanger sequencing)

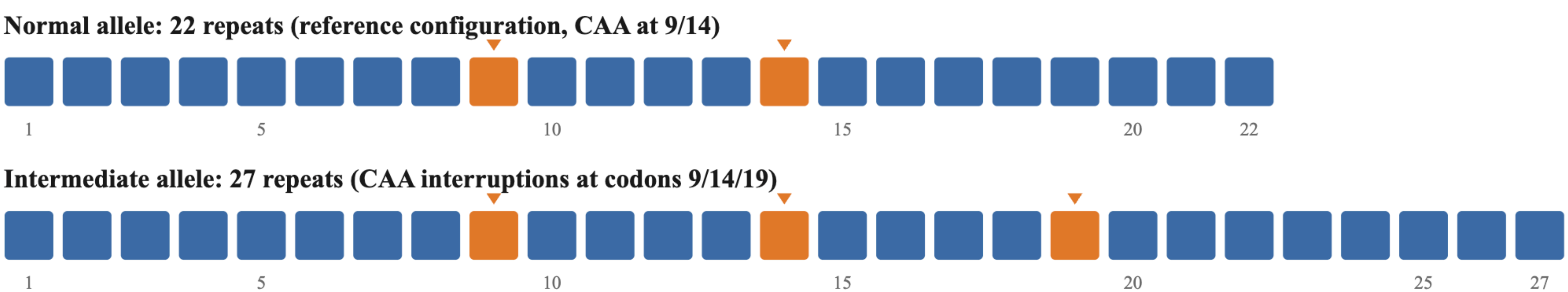


***$(CAG)_8$ · CAA · $(CAG)_4$ · CAA · $(CAG)_4$ · CAA · $(CAG)_8$***

*Identical configuration confirmed in both siblings; interrupted pattern characteristic of ALS-associated alleles.*

CAG

CAA (interruption)
