# Supplementary material for: Cerebellar ataxia-onset ALS with SOD1 D91A mutation: a rare phenotype
Source: Front Neurol. 2026 Aug 4;17:1889931. doi: 10.3389/fneur.2026.1889931 (PMC13481206; doi:10.3389/fneur.2026.1889931)
Supplement: Supplementary file 3 [file Table_1.docx]

**Supplementary Table S1.** Results of the diagnostic panel used to exclude alternative hereditary and acquired causes of progressive ataxia in the four patients.

| **Investigation** | **Case 1** | **Case 2** | **Case 3** | **Case 4** |
| --- | --- | --- | --- | --- |
| **Hereditary ataxias – repeat-expansion and gene-panel testing** | | | | |
| SCA1 (*ATXN1*) | Negative | Negative | Negative | Negative |
| SCA2 (*ATXN2*, full-length expansion) | Negative | Negative | Negative | Negative |
| SCA3 (*ATXN3*) | Negative | Negative | Negative | Negative |
| SCA6 (*CACNA1A*) | Negative | Negative | Negative | Negative |
| SCA7 (*ATXN7*) | Negative | Negative | Negative | Negative |
| SCA8 (*ATXN8/ATXN8OS*) | Negative | Negative | Negative | Negative |
| SCA17 (*TBP*) | Negative | Negative | Negative | Negative |
| Friedreich ataxia (*FXN*) | Negative | Negative | Negative | Negative |
| CANVAS (*RFC1*) | Negative | Negative | Negative | Negative |
| *C9orf72* (G4C2) | Negative | Negative | Negative | Negative |
| *ATXN2* CAG repeat sizing | 22/22 | 22/22 | 22/22 | **22/27 †** |
| **Acquired, metabolic and autoimmune causes** | | | | |
| Vitamin B12 (serum) | Normal | Normal | Normal | Normal |
| Folate (serum) | Normal | Normal | Normal | Normal |
| Vitamin E (serum) | Normal | Normal | Normal | Normal |
| Thyroid function (TSH, fT4) | Normal | Normal | Normal | Normal |
| Anti-gliadin antibodies | Negative | Negative | Negative | Negative |
| Anti-GAD antibodies | Negative | Negative | Negative | Negative |
| Paraneoplastic / onconeural antibodies | Negative | Negative | Negative | Negative |
| Neuronal surface (autoimmune) antibodies | Negative | Negative | Negative | Negative |
| CSF analysis | Unremarkable | Unremarkable | Unremarkable | **Mild ↑ protein (0.74 g/L)** |
| **Established molecular diagnosis** | | | | |
| *SOD1* c.272A>C (p.Asp91Ala) | **Heterozygous** | **Heterozygous** | **Homozygous** | **Homozygous** |

**†** Intermediate-length allele; Sanger sequencing confirmed 27 CAG repeats with CAA interruptions at codons 9, 14 and 19, identical in both siblings (see Supplementary Figure S2 A, B).

Negative, not detected / no pathogenic expansion; Normal, within the reference range. SCA2 testing detected no full-length (pathogenic) *ATXN2* expansion; intermediate-length alleles are reported in the dedicated *ATXN2* row.

The affected sister of Case 4 (homozygous *SOD1* D91A) was likewise negative for the *C9orf72* expansion and carried the identical *ATXN2* 22/27 allele.

Abbreviations: CANVAS, cerebellar ataxia, neuropathy and vestibular areflexia syndrome; CSF, cerebrospinal fluid; GAD, glutamic acid decarboxylase; SCA, spinocerebellar ataxia; TSH, thyroid-stimulating hormone; fT4, free thyroxine.
